# Christ Siemens Touraine syndrome: a case report

**DOI:** 10.1186/1757-1626-2-38

**Published:** 2009-01-10

**Authors:** Luiz Gutenberg TM Coelho, Arnaldo F Caldas, Evelyne P Soriano, Veronica MS Rodrigues, Roseane S Costa

**Affiliations:** 1 Department of Dental Public Health, University of Pernambuco, Avenida General Newton Cavalcanti, 1650, Camaragibe, Pernambuco, Zip Code: 54783-220, Brazil.

## Abstract

**Background:**

The ectodermal dysplasias are a large and complex group of diseases.

**Case presentation:**

This article presents a case in a 37 years old female patient, referred to the dental clinic for impairment patients maintained by the University of Pernambuco. She presented typical characteristics of Christ Siemens Touraine syndrome such as alterations of the inferior members, a great number of diffuse pigmentations, poor oral hygiene, periodontal disease, oligodontia, enamel hypoplasia, including alteration in the form and size of the teeth.

**Conclusion:**

The optimal treatment for these patients should require the multidisciplinary collaborative efforts of health professionals.

## Background

As defined and classified by Freire-Maia in 1971, ectodermal dysplasias may be subdivided into two groups: First, group A disorders, which are manifested by defects in at least 2 of the 4 classic ectodermal structures (trichodysplasias, dental abnormalities, nail abnormalities and dyshidrosis), mnemonically labeled 1, 2, 3 and 4, respectively, and second, group B disorders, which are manifested by defect signs in only one of the classic ectodermal structures above mentioned in combination with a defect in a different ectodermal structure such as ears, and lips. Eleven group A subgroups were defined in 1971, each with a distinct combination of 2 or more ectodermal defects. Today, more than 191 subgroups have been identified [1].

The most common form of Ectodermal Dysplasias (ED), the anhidrotic (or hypohidrotic) ectodermal dysplasia, a rare recessive genetic disease linked to chromosome X[2,3], is characterized by heat intolerance, excessively dry skin due to the absence of sweat glands and abnormal spiky or absent teeth. It is caused by mutation in a movel transmenbrane protein, ectodysplasin A,[4] mainly involving ectodermal structures such as epidermis and its annexes (hair and nails), although nonectodermal tissue may also become involved. Otolaryngological manifestations are related to hypoplasia of the mucous glands of the upper aerodigestive tract such as rhinitis, pharyngitis, bronchitis and otitis, and also epistaxis, dysphagia, anodontia and ozena, among others [2,5].

Among the ectodermal dysplasias, there are several examples of overlapping phenotypes in disorders that are considered distinct. In CST syndrome, dental care is frequently the most important aspect of the treatment required to improve and maintain mastigatory function and optimal facial appearance. Dental care is of great psychological benefit to syndromic patients, moreover when they have difficulties in finding dentists able to diagnose their problem and when the treatment means a financial burden for them and their families. In this case report we compare the findings in our patient with those in the literature.

## Case presentation

M.L.B.V, female, 37 years old, living in the countryside of the state of Pernambuco-Brazil, home-worker, self-related white ethnicity was referred to the dental clinic for patients with special needs of the University of Pernambuco.

The anamnesis showed she did not smoke or drinks alcohol, had normal somatic and mental development, excellent interaction and socialization degree. She also demonstrated cognitive capacity and oral language according to her age and socioeconomic level. She did not have any pregnancy. Her mother said her daughter had been diagnosed with ectodermal hypohidrotic dysplasia in childhood. No other cases of the disease in the family were acknowledged, though.

**Figure 1:**
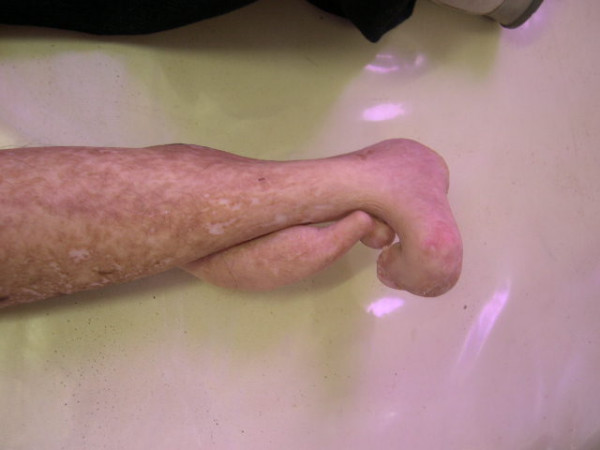
**Malformation of the right lower limb**.

**Figure 2:**
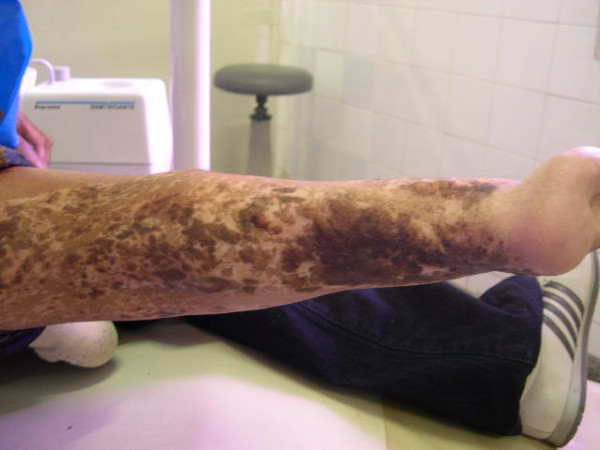
**Pigmented nevus at the lower limb**.

**Figure 3:**
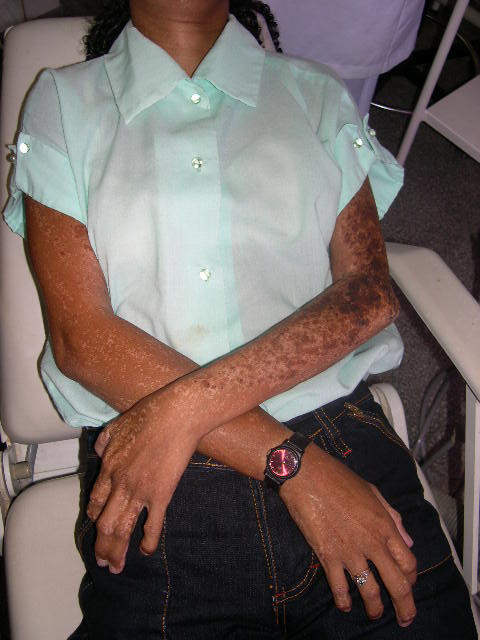
**Pigmented nevus at the arms and forearms**.

In the general physical exam, the patient presented height around 1,55 meters, low discreet weight, hypoplasia of mammas, locomotion difficulty due to malformation of the right lower limb (figure 1), pigmentation alteration all over the body, mainly in the upper and lower members, characterizing the pigmented nevus (figures 2 and 3), sindactilia of the left hand (figure 3) and hypohidrosis (decrease of sweating).

The craniomaxillofacial paraclinical approach showed a prominent forehead and an old-looking face, low-implanted ears with malformation of the left ear, nose in saddle, hypotrichosis (decrease in the amount of hair). In relation to the morfofunctional aspects, the patient presented mixed breathing (nasal and oral), atypical deglutition, facial asymmetry and deviation of nasal septum.

**Figure 4:**
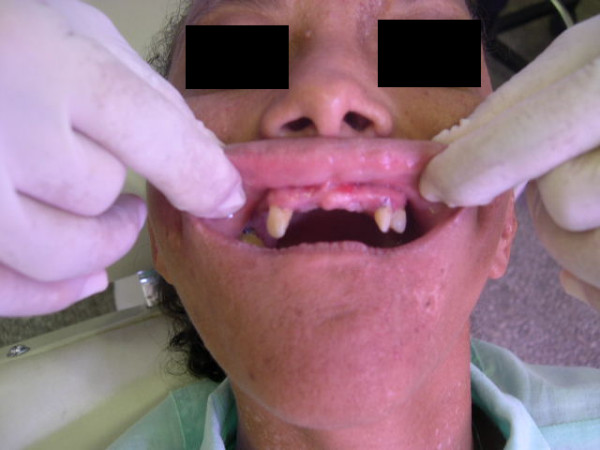
**Oral aspects of the patient**.

The intraoral examination showed poor oral hygiene, evaluated through the Simplified Index of Oral Hygiene (IOH-S = 4,0). Diffuse gingivitis, local periodontal disease, oligodontia, enamel hypoplasia and alteration in the form and size of the teeth were also observed (figure 4).

Therapeutics consisted in measures to restore the balance of oral microbiota such as antiseptic mouthwash for its chemical control, tooth brushing orientation, extraction of dental elements associated to periodontal disease, atraumatic restorative treatment, all with a view to prosthodontics rehabilitation. Now, at two-year follow-up, the patient's periodontal and dental health is under control.

## Discussion

Clinical diagnosis of ectodermal dysplasia is difficult. Identification of the precise syndrome would be a challenge for professionals without the patient's collaboration and the support of different specialties.

If the historical classification by Freire-Maia and Pinheiro and that by Priolo were strictly applied, the term "ectodermal dysplasias" would cover an endless number of diseases and would be useless for clinical practice.[6]

Although the most common form of ED is Christ-Siemens-Touraine syndrome, several other forms of EDs have clinical manifestations that affect the head and neck, particularly ectodactyly-ectodermal dysplasia-clefting syndrome (EEC) and ankyloblepharon-ectodermal defects-clefting syndrome (AEC, or Hay-Wells Syndrome), both including cleft lip and/or cleft palate [3].

Bokhoven et al [7] described LMS (limb-mammary syndrome), a new syndrome characterized by defects of the limbs, nipples, and mammary glands. The major differential diagnosis for LMS is probably the EEC syndrome. However, mammary gland and nipple defects, the most consistent features in this LMS family, have been reported only occasionally by patients with classic EEC. Furthermore, skin and hair anomalies have a very high incidence among patients with EEC but were not observed in any patient in this LMS family.

Though our female patient presented a severe manifestation of the Christ Siemens Touraine syndrome, the absence of congenital antecedents and consanguinity were of note. The anamnesis did not reveal the use of any abortive medication which could have caused the structural alteration of the lower limbs.

The presence of diffuse pigmentations suggested the possibility of Pigmentary Incontinence. According to Phan et al [8], Incontinentia Pigmenti (IP) or Bloch-Sulzberger syndrome is a rare X-linked dominant, multisystem neuroectodermal disorder which predominantly affects the skin, teeth, eyes, central nervous system (CNS), hair and nails.

Despite dermatological manifestations, all the characteristics observed in skull, face, hair, teeth, nails, skin, and sweat glands during physical examination as well as information concerning her family history and the absence of neurological involvement point to Christ Siemens Touraine Syndrome, associated to a specific malformation of the right lower limb.

The diagnosis of ED can be difficult due to a variety of types, range of abnormalities, and severity of defects. However, it is important to identify the components of the disorder so that appropriate treatment can be rendered to ED patients. It is also important to understand the genetic hereditary patterns so that the parents of an affected child can be advised on the possibility of new cases in the family [9].

## Conclusion

A multidisciplinary attention should be given to syndromic patients. Professionals should interact in providing them with an integral and effective treatment, mainly those of low socioeconomic condition. These patients' oral functions should be completely restored so that their harmonious reinsertion into society becomes feasible.

## Consent

Written inform consent was obtained from the patient for publication of this case report and accompanying images. A copy of the written consent is available for review by the Editor-in-chief of this journal.

The inform consent was sent by the patient's mother.

## Competing interests

The authors declare that they have no competing interests.

## Authors' contributions

VMSR and RSC: performed the dental treatment.

AFCJr, LGTMCJr and EPS: wrote the paper.
